# Multi-Informant Oral Diadochokinesis Assessment in Mandarin-Speaking Children and Adolescents: Protocol for a Cross-Sectional Observational Study

**DOI:** 10.2196/99571

**Published:** 2026-11-03

**Authors:** Guannan Wang, Shin Ying Chu, Siaw Chui Chai

**Affiliations:** 1 Centre for Healthy Ageing and Wellness, Faculty of Health Sciences, Universiti Kebangsaan Malaysia, Jalan Raja Muda Abdul Aziz, Kuala Lumpur, 50300, Malaysia, 60 3-9289-5023; 2 North Anhui Health Vocational College, Suzhou, Anhui, China; 3 Centre for Rehabilitation and Special Needs, Faculty of Health Sciences, Universiti Kebangsaan Malaysia, Kuala Lumpur, Malaysia

**Keywords:** adolescent, child, Mandarin, oral diadochokinesis, protocol, speech-language pathology, multi-informant assessment, audio recording, forced vital capacity, BMI

## Abstract

**Background:**

Oral diadochokinesis (oral-DDK) measures rapid, accurate repetition of syllables or syllable sequences and is widely used in speech motor assessment. Its interpretation requires attention to task demands and medical or physiological factors, as well as possible correspondence with everyday speech.

**Objective:**

This study aims to estimate task-, age-, and sex-related patterns in correct oral-DDK rate among Mandarin-speaking children and adolescents aged 8 to 17 years; examine associations with medical and physiological measurements; and assess correspondence with parent and teacher observations of everyday speech.

**Methods:**

A dedicated cross-sectional school study was conducted in Suzhou, Anhui Province, China, from October 2024 to June 2025. Schools were randomly selected, and homeroom teachers invited eligible students from all classes. The 7-stimulus battery comprised conventional alternating motion rate, nonword sequential motion rate (SMR), and Mandarin real-word SMR. All completed performances were digitally recorded. A total of 3 professional modules documented medical and physiological background, task readiness, and 5 oromotor actions. Physicians measured height, weight, and forced vital capacity, and BMI was calculated. Speech-language pathologists administered the readiness and oromotor checks. Readiness included a brief comprehension check using 5W1H (What, Why, Who, When, Where, and How) prompts. Primary guardians and homeroom teachers each completed 3 frequency items about inaccurate speech sounds and speech perceived as too fast or too slow. Eligibility and recording usability determined the analyzable observations. A repeated-task mixed model was used to estimate rate patterns; medical, physiological, and contextual report associations will be examined separately.

**Results:**

Field data collection ended in June 2025. Of 1036 participants, 2 (0.2%) withdrew, 6 (0.6%) experienced technical failures, and 2 (0.2%) could not complete the required screening or oral-DDK procedures; 1026 (99%) participants were retained. Oral-DDK scoring and verification and the primary rate analyses were completed by September 2026. As of October 2026, analyses linking rate to parent and teacher reports had not begun. A major revision of the normative manuscript was completed in early October 2026, but its publication timing is not yet known. This protocol does not present inferential results.

**Conclusions:**

The protocol links conventional and real-word oral-DDK with separately recorded health, physiological, and everyday speech observations. It specifies how these records enter analyses of correct rate and preserves a standardized digital audio corpus for scoring checks and subsequent task-specific analyses.

## Introduction

Oral diadochokinesis (oral-DDK) is a standard speech motor task that measures rapid syllable repetition. Alternating motion rate (AMR) uses a repeated syllable, whereas sequential motion rate (SMR) uses a syllable sequence. Fletcher [1] established time-by-count measurement of syllable rate. Kent et al [2] reviewed 360 oral and laryngeal DDK studies and documented substantial differences in elicitation, scoring, and reference populations. Pediatric research in Hebrew, Dutch, Thai, Cantonese, Korean, and Greek has examined how rate and accuracy vary with age and task [3-11]. Adult cross-language comparisons likewise caution against treating reference rates as interchangeable across linguistic settings [12-15].

Tseng and Jeng [16] studied real-word and nonword oral-DDK in Taiwanese preschoolers, and Jeng [17] subsequently included school-age children in a lifespan study. Chu et al [13] compared real-word and nonword tasks in Mandarin-speaking adults in Malaysia. To our knowledge, corresponding real-word evidence for children and adolescents recruited in mainland China remains lacking. This study examines task-, age-, and sex-related differences in correct oral-DDK rate among Mandarin-speaking children and adolescents aged 8 to 17 years. It also examines how medical and physiological measurements and parent and teacher observations of everyday speech relate to these rates.

Speech motor development is uneven: jaw control becomes more adult-like before lip control [18], and early speech movements differ from silent oral movements [19]. Smith’s [20] synthesis relates these changes to developing interactions between linguistic goals and motor control. More directly relevant to the present age range, Lancheros et al [21] found similar AMR and SMR rates in children aged 7 to 9 years, but faster SMR rates in adolescents and adults. We therefore analyze stimuli separately and estimate how task differences change with age.

When correct oral-DDK rate is low, a clinician may ask whether the child also makes frequent speech sound errors or speaks slowly in daily life. A high rate may prompt questions about speech perceived as too fast. The study records these observations systematically through parent and teacher reports. This attention to everyday functioning draws on the International Classification of Functioning, Disability and Health (ICF), which relates functioning to health and environmental conditions [22]. Its 2007 Children and Youth Version provided a child development perspective [23]. Bronfenbrenner [24] places child development in linked home, school, and community settings [25]. Pediatric surveillance attends to concerns from adults who know the child while distinguishing those observations from standardized screening and diagnosis [26].

Parents and teachers observe speech in settings that a brief examination cannot reproduce. Achenbach et al [27] examined cross-informant correlations in relation to situational specificity. De Los Reyes and Kazdin [28] distinguished contextual differences from differences in how informants retrieve and report information. A later meta-analysis of 341 mental health studies found mean cross-informant correlations of 0.25-0.30 [29]. This is a reason to examine disagreement, not a benchmark for validating speech report items. Speech-specific evidence comes from McLeod et al [30]: their 7-item Intelligibility in Context Scale correlated with clinical speech accuracy measures in 120 preschoolers, including an *r* of 0.54 for the percentage of consonants correct. That finding supports obtaining everyday observations while leaving the performance of the 3 items used here to be tested.

Task differences also limit what a rate can represent. In adults with motor speech disorders, Ziegler [31] found that DDK and sentence production were affected differently across syndromes. This does not settle the boundary between speech and nonspeech: Maas [32] challenged the definitions used to draw a sharp distinction. Lancheros et al [33] found slower sentence production and heterogeneous syllable DDK in adults with apraxia of speech, whereas a significant reduction was not detected for homogeneous repetition. These findings favor comparisons between specified tasks rather than treating all oral performance as a single ability.

Reviews distinguish the goals of speech and nonspeech oral tasks [34] and emphasize explicit task descriptions when considering their clinical uses [35]. Pediatric speech mechanism screening provides an observational precedent [36]. Here, the 5-action oromotor screen records visible performance to define eligibility.

The study retains conventional AMR syllables (/pa/, /ta/, and /ka/) and the nonword SMR sequence /pataka/, alongside Mandarin real-word SMR. Formative work included a review of candidate words by 10 experts in speech-language rehabilitation (n=5, 50%), linguistics (n=3, 30%), or both fields (n=2, 20%) and picture-naming familiarity testing in 55 children aged 3 to 5 years. Physicians reviewed the medical and physiological items with speech-language pathologists (SLPs), and the supervisory group reviewed the parent and teacher items. These steps shaped the field procedures described in the Methods section.

Published oral-DDK studies use different elicitation and timing conventions rather than one universal sampling duration [2]. Children must sustain articulatory repetition within the available breath. A companion study from the same doctoral cohort examined age, body size, and forced vital capacity (FVC) in relation to sustained /a/ and repeated /ka/ [37]. That study provides related evidence for considering respiratory capacity alongside the task being performed. Height, weight, and calculated BMI form the anthropometric group; FVC is considered separately. Health on the test day is also recorded.

The primary objective is to estimate task-, age-, and sex-related patterns in correct oral-DDK rate in Mandarin-speaking children and adolescents aged 8 to 17 years. Further objectives are to examine medical and physiological associations with rate and determine whether perceived fast or slow speech at home and school corresponds to examination performance. Readiness and 5-action oromotor criteria define the analysis population. Recordings are retained for subsequent accuracy analyses, including correspondence with the reported frequency of everyday speech sound errors.

## Methods

### Study Design and Protocol Status

This paper reports the protocol implemented in a cross-sectional observational study with completed field data collection. The ethics-approved school study retained standard oral-DDK and recorded parent, teacher, medical, physiological, readiness, oromotor, and audio information as separate sources.

The doctoral proposal established the procedures, substantive questions, and original recruitment calculation. The OSF Registries record [38] was deposited retrospectively after field data collection. Reporting follows the STROBE (Strengthening the Reporting of Observational Studies in Epidemiology) guidance for cross-sectional observational studies [39] (Checklist 1).

### Setting and Participants

Participants were recruited from mainstream schools in Suzhou, Anhui Province, China, between October 2024 and June 2025. Schools were selected at random from the citywide sampling frame. All classes in participating schools were included, and homeroom teachers invited all eligible students. Students who chose to participate completed the study forms after guardian consent and child assent. Assessments took place in quiet rooms on school premises. Physicians completed the medical and physiological measurements, SLPs administered the readiness and the 5-action oromotor screen, and trained medical student research assistants operated the recording workflow.

The target population comprised Mandarin-speaking children and adolescents aged 8 to 17 years who attended mainstream schools. Eligibility records confirmed that Putonghua was the only language used in everyday communication. Participant flow is reported in the Results and Figure 1; missing or technically unusable records within the retained cohort will be reported by task and source.

**Figure 1.**
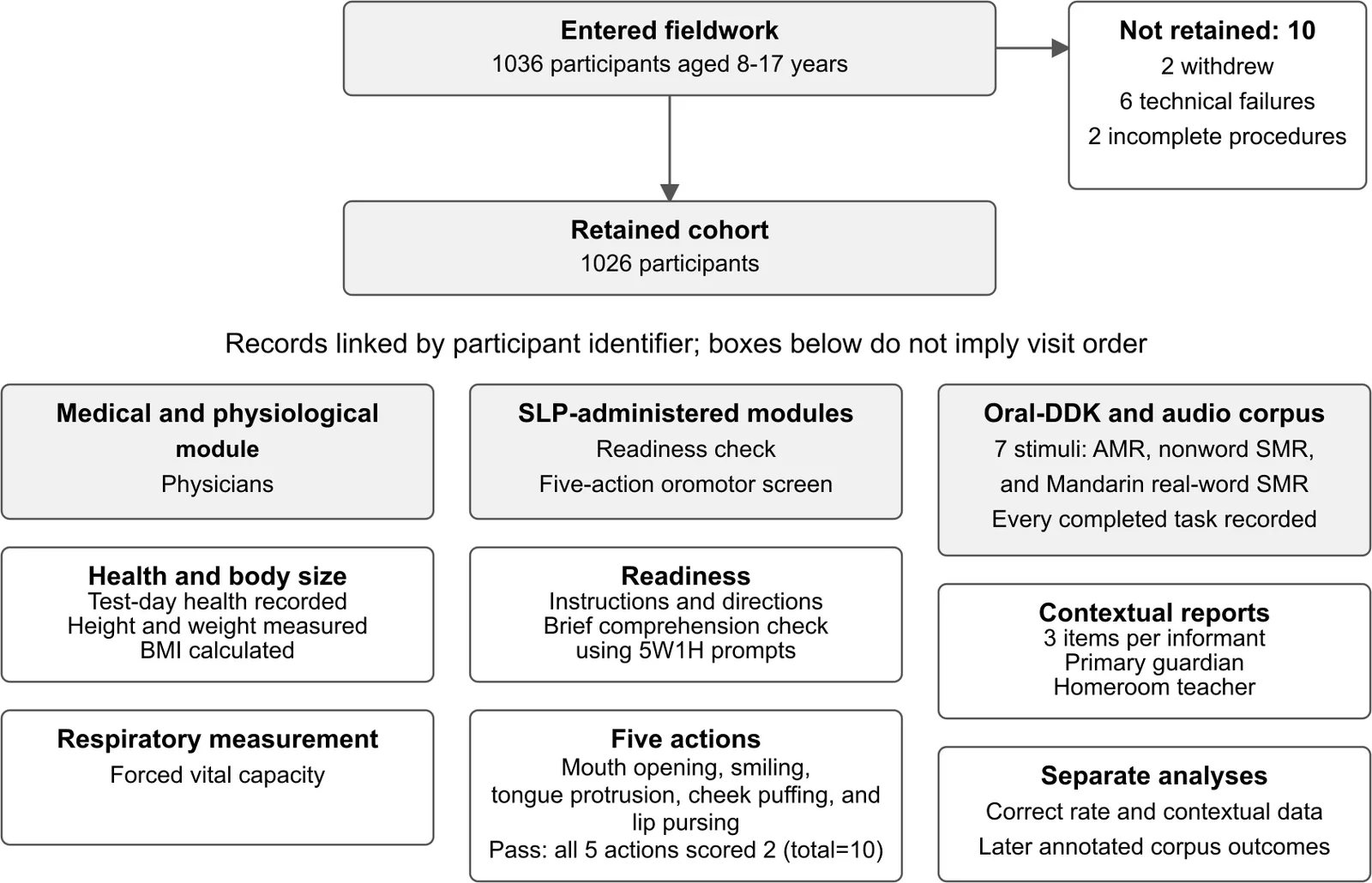
Participant flow and linked study records. The retained cohort comprised 1026 participants after 10 participants were not retained. The professional modules, oral diadochokinesis (oral-DDK) recordings, and home and classroom reports were linked by participant identifier. The lower panel shows sources of information, not their order within a visit. 5W1H: What, Why, Who, When, Where, and How; AMR: alternating motion rate; SLP: speech-language pathologist; SMR: sequential motion rate.

### Protocol Development

Development began with conventional AMR and SMR elicitation, digital recording, and count-with-time scoring [1,2]. Ten experts identified their primary expertise as speech-language rehabilitation (n=5), linguistics (n=3), or both fields (n=2). Their review narrowed the Mandarin real-word candidates. A picture-naming familiarity study with 55 children aged 3 to 5 years then tested whether children could produce the shortlisted words independently. This stimulus selection work was presented at Language Disorders 2026 Online.

Additional records were chosen because they could alter oral-DDK performance or its interpretation. The medical and physiological items underwent multidisciplinary review by 5 SLPs, 1 senior otorhinolaryngologist, 2 pediatricians, and 1 respiratory physician. The 5-action oromotor screen drew on established SLP practice and professional consultation. The readiness sequence adapted instruction-following procedures from structured child assessment. Operational rules for both SLP-administered checks are reported below.

The parent and teacher items arose from clinical follow-up questions used over more than a decade and were reviewed by the supervisory group. The same 3 items were collected from both sources to examine everyday speech alongside examination performance.

Before fieldwork, personnel completed 12 hours of structured training over 4 weeks. The team worked through each measurement and recording procedure and applied common administration and scoring criteria. During the final 2 weeks, 100 first-year medical students, who were not part of the pediatric cohort, served as a practice sample for standardizing workflow and examiner behavior.

### Parent and Teacher Contextual Reports

Primary guardians reported home communication, and homeroom teachers who had known the child for at least 6 months reported classroom communication. Both rated typical behavior during the preceding month on the same 3 items: inaccurate speech sounds, speech that seemed too fast, and speech that seemed too slow. Responses were ordered frequency categories: 0=never, 1=occasionally, 2=often, and 3=always. Instructions required mutually exclusive responses for the too fast and too slow items; any contrary entries were flagged as invalid. If neither direction applied, both were 0. The questions ask what adults repeatedly noticed, not whether the child has a disorder or how the child performed oral-DDK.

The protocol uses 3 prespecified oral-DDK contextual items from each source, reproduced in Multimedia Appendices 1 and 2. Analyses remain item-specific, with no composite score.

Analysis will first describe each item’s distribution, missingness, and floor or ceiling pattern. Parent-teacher agreement will be reported item by item using raw agreement and weighted κ; Gwet AC2 will be added when sparse categories or floor effects make κ unstable. These are cross-informant comparisons, not repeated measurements by the same rater. The current 8 to 17-year-old cohort did not undergo a separate report item validation study or test-retest assessment.

Dual nonzero responses to the mutually exclusive fast and slow items will be treated as invalid for the rate-direction analysis and reported. Fast and slow observations will be tested separately against age-, sex-, and task-adjusted oral-DDK rate. Estimates will be reported regardless of direction or statistical significance. If an item has no variation, its distribution will be reported, and associations that cannot be estimated will be identified as such. The item on inaccurate speech sounds is reserved for the corresponding accuracy analysis. These checks evaluate the behavior of contextual report variables; they do not convert the forms into diagnostic speech proficiency scales.

### Three Professional Modules and Field Roles

Physicians administered the medical and physiological module, and SLPs administered the readiness module and 5-action oromotor screen. Multimedia Appendix 3 [37] summarizes their field roles, administration procedures, and scoring rules.

An SLP administered a brief comprehension check using 5W1H prompts covering 6 question types: What, Why, Who, When, Where, and How. The team developed the questions, accompanying pictures, and predefined answer criteria, which were used consistently for all participants. The pictures served as illustrative aids for simple questions about familiar everyday situations. Passing required correct responses to all 6 question types. If a question was not understood, it could be repeated once verbatim, without hints, explanations, or rephrasing.

The 5 actions were mouth opening, smiling, tongue protrusion, cheek puffing, and lip rounding or pursing. A score of 0 meant that the child could not complete the action; 1 meant completion with an observable limitation in range, symmetry, stability, or seal, as applicable; and 2 meant completion without an observable quality limitation under the field instructions. Passing required a score of 2 on each action (total=10).

During the on-site examination, physicians judged and separately coded acute infection, chronic infection, and current ear, eye, or nasal inflammation (0=absent, 1=present). They measured height and weight; BMI was calculated as weight (kg)/height (m)^2^. FVC was measured twice in the standing position, following the device instructions and the same procedure as the companion study [37]. Anthropometry and FVC were treated as separate groups in the analysis.

### Oral-DDK Task Battery and Administration

The battery comprised 7 stimuli. AMR comprised /pa/, /ta/, and /ka/; nonword SMR comprised /pataka/; and Mandarin real-word SMR comprised 怕它卡 /pa4 ta1 ka3/, 怕他看 /pa4 ta1 kan4/, and 葡萄干 /pu2 tao2 gan1/. Testing followed a fixed block order—AMR, nonword SMR, then real-word SMR—with item order randomized within the AMR and real-word blocks.

A brief /ka/ pretest familiarized the child with rapid repetition. For each formal stimulus, the examiner gave 1 demonstration and the child completed 1 unscored imitation; no additional formal practice was provided. After the cue, the child took a deep breath and repeated the target as quickly, accurately, and continuously as possible. One formal production was planned per stimulus. A task was repeated only after a procedural or technical validity problem; a valid but slow performance was not repeated. At least 2 minutes separated blocks, and approximately 3 minutes preceded a required repeat.

### Digital Recording and Audio-Based Oral-DDK Scoring

All completed oral-DDK performances under the study protocol were digitally recorded. Recordings were made in quiet school rooms with an omnidirectional microphone approximately 8 to 10 cm from the child’s mouth, routed through a Roland QUAD-CAPTURE audio interface (UA-55). Audacity stored the working recordings in its native project format (.aup3); WAV copies were used for scoring. Password-protected local files used participant codes and stimulus identifiers. Multimedia Appendix 4 provides the recording and scoring procedures.

To calculate correct oral-DDK rate (syllables/s), we multiplied credited tokens by the number of syllables per token and divided the result by the analyzed breath trial duration. The interval began at the acoustic onset of the first credited production and ended at the offset of the last production in that breath trial; a pause longer than 0.2 seconds ended the current analysis interval. The demonstration and unscored imitation were excluded. For monosyllables, a token was credited when the target initial consonant was correct. Trisyllabic stimuli were credited only as fully correct whole tokens; no partial credit was assigned. The analyzed duration included time occupied by subsequent uncredited productions. Two trained medical student raters independently scored every recording by listening and waveform inspection. One SLP audited a random 10% as a third review. Training records documented task-level intraclass correlation coefficients above 0.90; analytic-sample agreement will be reported by task with 95% CIs.

### Study Variables and Measures

Table 1 summarizes the recorded variables, their sources, and their analytic roles.

**Table 1.** Study variables and measures.

Domain	Variables	Source	Scale	Analytic role
Background	Age, sex, and school level	Study record	Numeric or categorical	Age and sex in core model; school level descriptive
Readiness	Instruction understanding, direction-following, and brief comprehension check using 5W1H^a^ prompts	SLP^b^	Pass or exclude	Child eligibility
Health on the test day	Acute and chronic infection and ear, eye, or nasal inflammation	Physician	Binary	Separate health comparison
Body size	Measured height and weight and calculated BMI	Physician	Continuous or derived	Height and weight vs BMI alone
Respiratory physiology	FVC^c^	Physician	Continuous	Separate FVC comparison
Home context	3 observations of speech during the preceding month	Primary guardian	Ordinal	Item-specific home analysis
Classroom context	3 corresponding observations during the preceding month	Homeroom teacher	Ordinal	Item-specific classroom analysis
Five-action oromotor screen	Mouth opening, smiling, tongue protrusion, cheek puffing, and lip rounding or pursing	SLP	0‐2 per action	Child eligibility; all 5 actions must score 2
Core oral-DDK^d^	Correct oral-DDK rate for each of 7 stimuli	Trained medical student research assistants	Rate in syllables/s	Primary outcome
Corpus extension	Temporal and intensity variability, speech sound accuracy, and other acoustic measures	Stored audio	Derived	Later analyses

a 5W1H: What, Why, Who, When, Where, and How.

b SLP: speech-language pathologist.

c FVC: forced vital capacity.

d oral-DDK: oral diadochokinesis.

### Sample Size Justification

The doctoral proposal specified an a priori analysis of covariance calculation with Cohen *f*=0.15 [40], α=.05, and power=0.95. G*Power [41] yielded a required sample size of 580. A 10% allowance increased the target to 638, which was rounded to approximately 660. This calculation established the recruitment target specified in the original doctoral proposal. The present analysis uses the available cohort of 1026 and a repeated-stimulus mixed model; the original calculation does not establish power for that model or for each secondary association. Results will report model-specific sample sizes, effect estimates, and CIs.

### Statistical Analysis Plan

Figure 2 shows how the records enter analysis. Child-level readiness and oromotor criteria are applied before task-level recording usability and correct rate estimation. The core model estimates task, age, and sex patterns. Physician records and home and classroom observations are then examined in separate comparisons.

**Figure 2.**
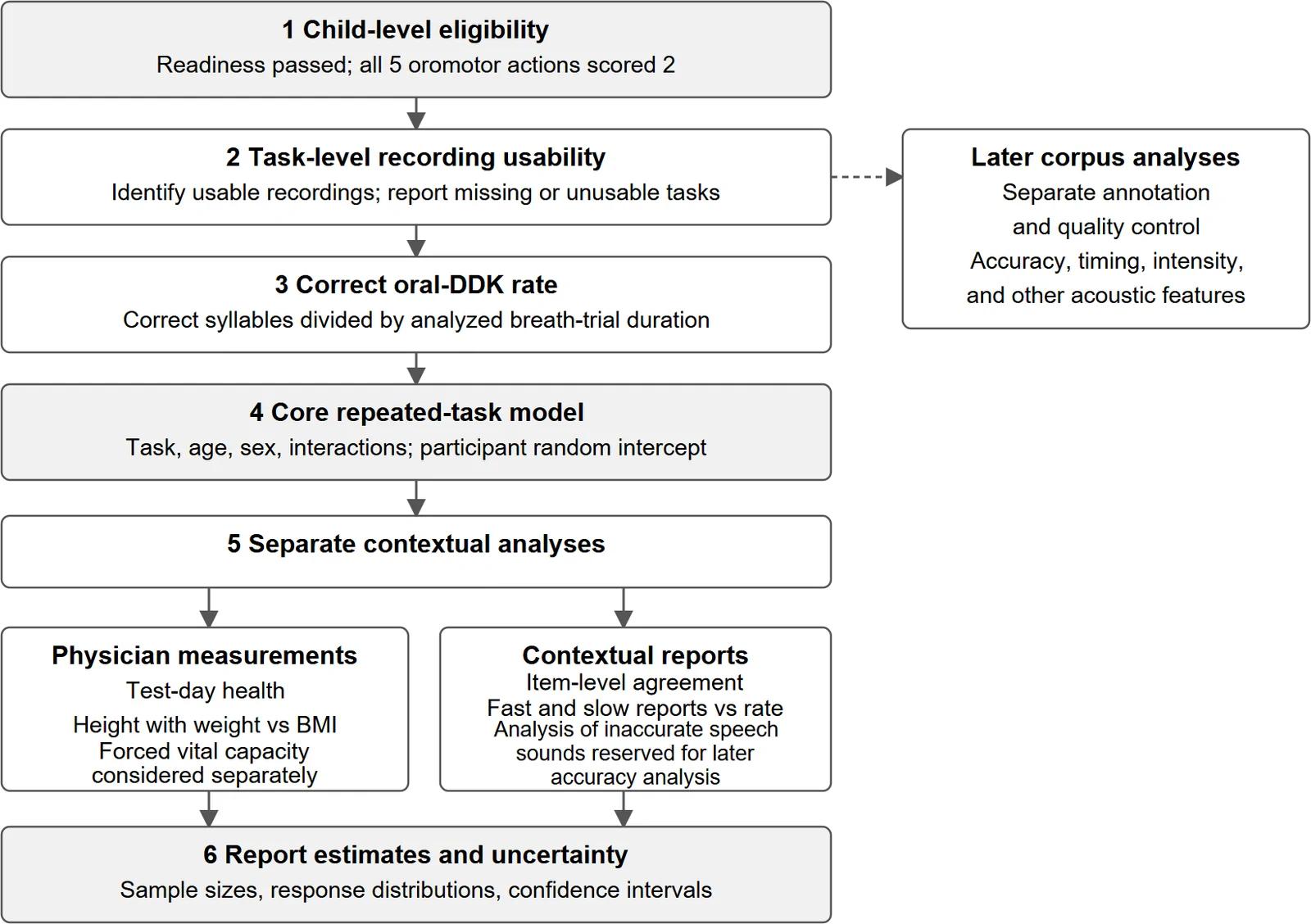
Analysis of linked study records. Readiness and the 5-action oromotor screen define child-level eligibility, followed by task-level recording usability and correct rate estimation. Analyses of medical and physiological measurements and contextual reports address separate questions around the core rate model. Arrows denote analytic dependencies, not causal effects. FVC: forced vital capacity; oral-DDK: oral diadochokinesis.

Descriptive analyses will report the participant flow shown in the Results and Figure 1, then give a separate usable denominator for every model. Continuous variables will be summarized with means and SDs or medians and IQRs; categorical variables will be summarized with counts and percentages. Missing or technically unusable task records will not be imputed. Their number and documented reason will be shown by stimulus, age band, and sex; remaining missingness will also be reported by source.

The primary outcome is audio-based correct oral-DDK rate. The core linear mixed-effects model includes a participant random intercept and fixed effects for task, centered age, sex, task-by-age, task-by-sex, age-by-sex, and task-by-age-by-sex. Readiness and oromotor results define eligibility rather than act as ordinary exposures. Same-day health, anthropometry, and FVC will then be added in separate comparisons. The anthropometric analysis will compare height with weight against BMI alone; all 3 will not enter one model together.

Model comparisons will report effect estimates, CIs, explained variation, and information criteria. Each parent or teacher item is recorded once per child and linked to that child’s stimulus-specific rates; the report is not a separate independent observation for each stimulus. Parent-teacher agreement will be reported item by item. Associations between fast or slow observations and adjusted oral-DDK rate will use proportional-odds ordinal models, with a rank-based sensitivity analysis if the proportional-odds assumption is not met. The item on inaccurate speech sounds will be analyzed with the later accuracy outcome.

Benjamini-Hochberg false discovery rate control will be applied within each source-specific family of secondary tests. Table 2 summarizes the analyses reported in this protocol.

**Table 2.** Analyses specified in this protocol.

Analysis question	Variables	Method	Role
Task, age, and sex patterns	Correct oral-DDK^a^ rate	Mixed model with fixed effects for task, centered age, sex, and all 2- and 3-way interactions, and a participant random intercept	Primary analysis
Readiness	Instruction understanding, direction-following, and 5W1H^b^ check	Apply eligibility criteria before rate analysis	Child eligibility
Health on the test day	5 binary physician records	Descriptive and sensitivity models	Health context
Anthropometry	Height, weight, and BMI	Height and weight vs BMI alone	Body size context
Respiratory physiology	FVC^c^	Separate nested comparison	Respiratory context
Five-action oromotor screen	5 action scores	Apply eligibility criteria and describe the distribution of scores for each action	All actions must score 2 (total=10)
Home and classroom correspondence	3 parallel items per source	Raw agreement, weighted κ, and Gwet AC2 if sparse categories destabilize κ	Cross-informant convergence
Speech rate observations	Fast and slow items and adjusted oral-DDK rate	Proportional-odds models and rank-based sensitivity analysis if assumptions fail	Directional association
Contribution of added records	Model fit and explained variation	Nested comparisons, information criteria, estimates, and CIs	Source-specific contribution
Report item behavior	Missingness, floor effects, and ceiling patterns	Descriptive item-level checks	Contextual measurement checks
Corpus extension	Temporal and intensity variability, speech sound accuracy, and other acoustic measures	Separate preparation and analysis	Later work

a oral-DDK: oral diadochokinesis.

b 5W1H: What, Why, Who, When, Where, and How.

c FVC: forced vital capacity.

### Ethical Considerations

The study was approved by the Research Ethics Committee of Universiti Kebangsaan Malaysia (JEP-2024‐770) and the Suzhou First People’s Hospital Ethics Committee (SZYYLLky2024010). Written informed consent was obtained from parents or legal guardians, and assent was obtained from participating children before participation. Data were managed using coded participant identifiers. Access to identifiable data was restricted to authorized research personnel according to institutional procedures. Participating children received certificates of recognition only; no cash payments were provided.

### Data Management and Quality Assurance

Quality checks cover duplicate identifiers, impossible values, contradictions across records, missing data, audio file matches, and ambiguous counts. Audio is the source record for oral-DDK scoring; disagreements are resolved according to the scoring rules described above.

## Results

Recruitment and field data collection took place from October 2024 to June 2025. A total of 1036 children and adolescents aged 8 to 17 years entered fieldwork: 2 (0.2%) withdrew, 6 (0.6%) had technical failures, and 2 (0.2%) could not complete the required screening or oral-DDK procedures. The remaining 1026 (99%) participants were retained (Figure 1).

By September 2026, oral-DDK scoring and verification and the primary analyses of task-, age-, and sex-related rate patterns had been completed. A major revision of the normative manuscript was completed in early October 2026, but its publication timing is not yet known. As of October 2026, analyses linking oral-DDK rate to parent and teacher reports had not begun. A companion analysis of duration during a single breath and FVC from the same cohort was published in August 2026 [37]. This protocol documents the procedures and planned analyses without presenting their inferential results.

## Discussion

### Principal Features of the Protocol

The protocol links a defined rapid repetition task with records that address different aspects of its interpretation. Medical and physiological measurements are examined in relation to correct rate. Readiness and observable oromotor performance define the analysis population, while home and classroom reports document everyday speech that the examination does not directly sample.

The parent and teacher items formalize brief clinical follow-up questions. McLeod et al [30] found that everyday judgments related to clinical speech accuracy measures when assessed with a purpose-built instrument in preschool children. Their Intelligibility in Context Scale measures how readily different listeners understand the child; the present items record the frequency of perceived sound inaccuracy and rate direction. Reports of frequent speech sound errors may be informative even when adults do not perceive unusual speaking speed. Their correspondence with recorded accuracy will be examined separately.

Oral-DDK asks children to repeat specified syllables as rapidly and accurately as possible [2]. Everyday communication does not routinely require this maximum repetition rate. A child who achieves a high rate during testing need not speak rapidly in ordinary conversation. Correspondence between oral-DDK performance and everyday speech may therefore be incomplete. Weak associations could reflect limited correspondence between the two, restricted variation in the report items, or measurement error. These possibilities will be considered alongside the observed response distributions.

### Relation to Prior Work

Real-word stimuli do not consistently yield faster DDK rates than nonword stimuli. Tseng and Jeng [16] found no general rate advantage for the Mandarin sequence 怕他看 over /pataka/ in Taiwanese preschoolers. Among Mandarin-speaking adults in Malaysia, Chu et al [13] reported a higher mean rate for the English real-word stimulus than for /pataka/, but a lower mean rate for the Mandarin real-word stimulus. Lexical content, sound structure, and familiarity therefore need to be considered together. Retaining conventional tasks alongside the selected Mandarin words permits a direct task comparison in the mainland school cohort.

In a companion analysis of the same doctoral cohort, Wang et al [37] examined 1-breath endurance during sustained /a/ and repeated /ka/ in 971 participants with FVC measurements. The remaining 55 participants lacked FVC values. Age and FVC predicted endurance in both tasks. The association with FVC was stronger for sustained /a/ than for repeated /ka/, whereas the association with age was stronger for repeated /ka/. These task differences provide relevant evidence for considering respiratory capacity alongside oral-DDK performance. The present protocol examines the association of FVC with correct repetition rate rather than 1-breath duration.

Correct rate is the primary outcome specified in this protocol. Temporal and intensity variability, accuracy, and other acoustic features require further segmentation and annotation. Rate cannot describe all clinically relevant performance, particularly speech sound errors or irregular timing [2,3,8,9]. Retaining every completed recording allows these questions to be examined in the same productions when the additional preparation is complete.

### Strengths and Limitations

The study combines a defined 7-stimulus battery with independent dual scoring; a random 10% SLP audit; and separate physician, parent, and teacher records. The retained recordings permit scoring decisions to be checked. Keeping the information sources separate also makes it possible to report where their findings agree and where they do not.

Several design features limit interpretation. Only children meeting the readiness and 5-action criteria enter the primary rate analysis, so the findings cannot characterize those who fail these checks. The 5 actions do not constitute a comprehensive oral-mechanism examination. The six 5W1H question types and administration rules are reported, but the study-developed questions and answer keys are not supplied, limiting exact item-level replication of this readiness check. Exclusive use of Putonghua was established from eligibility records rather than a formal language exposure instrument, and the cohort represents mainstream schools in 1 region.

The contextual items lack independent validation and test-retest evidence in this age group. Missing and technically unusable records may change the samples contributing to individual analyses. The original recruitment calculation does not establish power for every interaction or secondary association. Finally, within-cohort associations cannot establish that the expanded protocol improves clinical decisions or outperforms assessment without the added records.

### Conclusions

This protocol documents conventional and Mandarin real-word oral-DDK tasks, linked professional assessments, and parent and teacher observations in a school cohort. It specifies how correct rate is scored and how medical, physiological, and everyday speech records enter separate analyses. The standardized digital audio corpus preserves the performances for scoring checks and subsequent task-specific analyses.

## Supplementary material

10.2196/99571 Multimedia Appendix 1 Parent contextual report items used in the present oral diadochokinesis analysis (bilingual English-Chinese).

10.2196/99571 Multimedia Appendix 2 Teacher contextual report items used in the present oral diadochokinesis analysis (bilingual English-Chinese).

10.2196/99571 Multimedia Appendix 3 Professional roles and 3-module field protocol (bilingual English-Chinese).

10.2196/99571 Multimedia Appendix 4 Professional audio recording and audio-based scoring rules (bilingual English-Chinese).

10.2196/99571 Checklist 1 STROBE checklist for a cross-sectional study.

## Abbreviations

5W1H: What, Why, Who, When, Where, and How
AMR: alternating motion rate
FVC: forced vital capacity
ICF: International Classification of Functioning, Disability and Health
oral-DDK: oral diadochokinesis
SLP: speech-language pathologist
SMR: sequential motion rate
STROBE: Strengthening the Reporting of Observational Studies in Epidemiology
