# Supplementary material for: Multi-Informant Oral Diadochokinesis Assessment in Mandarin-Speaking Children and Adolescents: Protocol for a Cross-Sectional Observational Study
Source: JMIR Res Protoc. 2026 Nov 3;15:e99571. doi: 10.2196/99571 (PMC13730412; doi:10.2196/99571)
Supplement: Supplementary file 2 [file resprot-v15-e99571-s002.pdf]

Teacher Contextual Report Items

教师情境报告条目（中英双语）

This appendix lists the 3 prespecified contextual report items collected alongside oral diadochokinesis (oral-DDK). The respondent was the child's homeroom teacher, who had known the child for at least 6 months and rated typical classroom communication during the preceding month. The Chinese items are accompanied by English translations. The sound-inaccuracy item formalizes a clinical follow-up question about everyday pronunciation. Items are analyzed separately without a composite score. Fast and slow speech items enter the rate analyses; inaccurate speech sounds are retained for subsequent accuracy analyses.

本附录列出伴随口腔轮替运动（oral-DDK）采集的 3 个预先设定情境报告条目。填写者为认识该儿童至少 6 个月的班主任，根据过去 1 个月儿童在课堂交流中的典型表现作答。中文条目附有英文翻译。发音不准条目将临床中对日常发音的追问固定为记录项目。各条目分别分析，不合成为综合得分。语速过快和过慢条目进入速率分析；发音不准条目保留用于后续准确性分析。

Response options / 反应选项

0=Never; 1=Occasionally; 2=Often; 3=Always. These are ordered frequency categories, not an equal-interval scale.

0=从不；1=偶尔；2=经常；3=总是。这些分值表示有序频率类别，不作为等距量尺。

Table S1. Contextual report items / 情境报告条目

**Item**<br>**条目**	**0**	**1**	**2**	**3**
Inaccurate speech sounds (eg, substitutions)<br>发音不准（如“哥哥”发成“蝈蝈”）	☐	☐	☐	☐
Speech rate is too fast<br>语速过快	☐	☐	☐	☐
Speech rate is too slow<br>语速过慢	☐	☐	☐	☐

Instructions required mutually exclusive responses for the two rate-direction items. Nonzero ratings for both fast and slow speech were flagged as invalid for rate-direction analyses only; other valid items were retained. If neither direction applied, both items were 0.

填写说明要求两个语速方向互斥。过快和过慢均为非零的记录，仅在语速方向分析中标记为无效；其他有效条目保留。若两个方向均不适用，则两项均为 0。

Measurement checks / 测量表现核查

By September 2026, analyses of associations between these reports and oral-DDK rate had not begun. Planned checks cover item distributions, missingness, floor and ceiling patterns, and item-specific parent-teacher agreement. Associations of the rate-direction items with adjusted correct oral-DDK rate will be reported with uncertainty, including weak, absent, or opposite-direction associations. If an item has no variation, its distribution will be reported and the association identified as not estimable. Parent-teacher agreement reflects correspondence across settings and informants; these items had not undergone independent validation, and no test-retest data were collected for these reports in this cohort. These checks evaluate the behavior of contextual report variables; they do not convert the forms into diagnostic speech-proficiency scales.

截至 2026 年 9 月，这些报告与 oral-DDK 速率的关联分析尚未开始。计划核查条目分布、缺失、地板与天花板效应及逐条目的家长与教师一致性。语速方向条目与调整后正确 oral-DDK 速率的关联将连同不确定性报告，包括较弱、未见关联或方向相反的结果。条目没有变异时，报告其频数分布，并说明关联不可估计。家长与教师一致性反映跨场景和跨信息源的对应；这些条目未在本队列开展独立验证，也未采集重测资料。这些核查用于评价情境报告变量的表现，不会把表格转化为诊断性言语能力量表。
