# Supplementary material for: Multi-Informant Oral Diadochokinesis Assessment in Mandarin-Speaking Children and Adolescents: Protocol for a Cross-Sectional Observational Study
Source: JMIR Res Protoc. 2026 Nov 3;15:e99571. doi: 10.2196/99571 (PMC13730412; doi:10.2196/99571)
Supplement: Supplementary file 3 [file resprot-v15-e99571-s003.pdf]

Professional Roles and Field Protocol

专业人员分工与三模块现场方案（中英双语）

Physicians completed the medical/physiological background module. Speech-language pathologists (SLPs) administered the readiness module and five-action oromotor screen. Trained medical-student research assistants recorded oral diadochokinesis (oral-DDK) separately. Readiness and oromotor results determine eligibility for rate analysis, rather than serving as predictors among children who passed. Physician measurements enter source-specific comparisons. Child participants received certificates of recognition only; no cash rewards were provided.

医生完成医疗/生理背景模块；言语语言治疗师（SLP）实施任务准备度模块及五动作口部运动筛查。经过培训的医学生研究助理另行执行口腔轮替运动（oral-DDK）录音。准备度与口部运动筛查结果用于确定速率分析资格，不作为通过者模型中的预测变量。医生测量项目按来源分别进入比较。参与研究的儿童仅获得表彰奖状，未获现金奖励。

Medical and physiological background / 医疗与生理背景

During the on-site examination, physicians judged acute infection, chronic infection, and current ear, eye, or nasal inflammation, each coded 0=absent or 1=present. Height and weight were measured; body mass index (BMI) was calculated from these measurements. Forced vital capacity (FVC) was measured twice in the standing position, following the device instructions and the same procedure as the companion study [1].

医生在现场检查中判断急性感染、慢性感染及当前耳、眼或鼻部炎症，各按 0=无、1=有编码。身高和体重由实测获得；体质指数（BMI）据此计算。用力肺活量（FVC）依照仪器使用说明，采用站立位测量 2 次，程序与同源研究一致[1]。

Table S1. Physician measurements / 医生测量项目

**Variable**<br>**变量**	**Unit or derivation**<br>**单位或计算**	**Analytic group**<br>**分析组**
Height<br>身高	Measured in cm<br>实测，单位为 cm	Anthropometry<br>体格
Weight<br>体重	Measured in kg<br>实测，单位为 kg	Anthropometry<br>体格
BMI<br>体质指数	Weight (kg) / height (m)², using measured values; unit: kg/m²<br>由实测体重（kg）除以身高（m）的平方计算；单位为 kg/m²	Anthropometry<br>体格
FVC<br>用力肺活量	Measured in mL<br>实测，单位为 mL	Respiratory physiology<br>呼吸生理

Examiner-administered readiness / 检查者实施的任务准备度

Readiness covers instruction understanding and direction-following. The research team developed the materials for this study-specific readiness check. The brief comprehension check using 5W1H prompts comprises 6 picture-supported question types: What, Why, Who, When, Where, and How. Every child received the same pictures and fixed questions, scored against a predefined answer key. The pictures served as illustrative aids to the questions rather than as targets for picture identification. The SLP scored each response as correct or incorrect. No hints, explanations, or rephrasing were allowed. If the child did not understand a question, the examiner could repeat it once verbatim. All 6 question types had to be answered correctly. This appendix provides the procedure for the six question types, scoring, and pass rule. The actual pictures, question wording, and answer key are not reproduced. The undisclosed wording and answer key limit exact item-level replication of this readiness check.

准备度包括指令理解与执行。这项研究专用准备度检查的材料由研究团队自行制作。使用 5W1H 提示进行的简短理解核查包含 What、Why、Who、When、Where 和 How 六种图片支持问题。所有儿童均使用统一图片、固定问题，并按预先确定的答案标准评分。图片用于辅助呈现问题，而非图片辨认的测试对象。SLP 将每个回答判为正确或错误。不允许提示、解释或改述。如果儿童没有听懂问题，检查者可原样重复 1 次。六种问题均须回答正确。本附录提供六种问题的施测程序、评分及通过规则。具体图片、题干及答案材料未在此复现；其中，未公开的题干和答案标准限制了对该准备度检查的逐项精确复现。

Five-action oromotor screen / 五动作口部运动筛查

The five actions are mouth opening, smiling, tongue protrusion, cheek puffing, and lip rounding/pursing. A score of 0 means unable to complete the action; 1 means completed with an observable limitation in range, symmetry, stability, or seal, as applicable; and 2 means completed without an observable quality limitation under the field instructions. Passing requires 2 on all 5 actions (total=10).

五个动作是张口、微笑、吐舌、鼓腮和嘟唇。0 分表示不能完成动作；1 分表示能够完成，但按动作特点存在可观察的幅度、对称性、稳定性或闭合限制；2 分表示按现场指令完成且未见可观察的质量限制。5 项均得 2 分、总分 10 分时通过。

References

1. Wang G, Fadzila NHM, Lee J, Jiang H, Chai SC, Chu SY, Barlow SM. Clinical relevance of maximum phonation time in children and adolescents: task-dependent roles of age, sex, and forced vital capacity. Clin Linguist Phon. 2026 Aug 11:1-20. PMID:42578533 doi:10.1080/02699206.2026.2713451
