# Supplementary material for: Multi-Informant Oral Diadochokinesis Assessment in Mandarin-Speaking Children and Adolescents: Protocol for a Cross-Sectional Observational Study
Source: JMIR Res Protoc. 2026 Nov 3;15:e99571. doi: 10.2196/99571 (PMC13730412; doi:10.2196/99571)
Supplement: Supplementary file 4 [file resprot-v15-e99571-s004.pdf]

## Digital Audio Recording and Scoring

数字录音与基于音频的计分规则（中英双语）

This appendix specifies the 7 oral diadochokinesis (oral-DDK) stimuli, administration sequence, recording files, analyzed interval, and scoring review. The same standardized digital audio corpus will support later temporal, intensity, accuracy, severity, and other acoustic analyses after separate segmentation, annotation, quality control, and analytic verification.

本附录列出 7 项口腔轮替运动（oral-DDK）刺激、施测顺序、录音文件、分析区间及评分复核。同一标准化数字音频语料库将在另行完成切分、标注、质量控制和分析验证后，用于时间、强度、准确性、严重度及其他声学分析。

### Digital audio recording / 数字录音

All completed oral-DDK performances under the study protocol were digitally recorded. Trained medical-student research assistants operated the recording workflow.

本研究方案下所有已完成的 oral-DDK 表现均进行数字录音。录音由经过培训的医学生研究助理执行。

**Table S1. Recording equipment and files / 录音设备与文件**

| **Parameter**<br>**参数** | **Protocol specification**<br>**方案设定** |
|---|---|
| Recording environment<br>录音环境 | Quiet school-based rooms<br>安静的校内房间 |
| Microphone position<br>麦克风位置 | Omnidirectional microphone approximately 8-10 cm from the participant’s mouth<br>全向麦克风置于距参与者口部约 8-10 厘米处 |
| Audio interface<br>声卡 | Roland QUAD-CAPTURE audio interface (UA-55)<br>Roland QUAD-CAPTURE 音频接口（UA-55） |
| Software and files<br>软件与文件 | Audacity native project storage (.aup3); Waveform Audio File Format (WAV) copies for scoring<br>Audacity 原生项目文件（.aup3）；评分使用 WAV 副本 |
| File tracing<br>文件追踪 | Participant codes and stimulus identifiers<br>参与者编码和刺激标识 |

**Task battery and administration / 任务与施测**

Seven stimuli were administered in a fixed block order: alternating motion rate (AMR; /pa/, /ta/, /ka/), nonword sequential motion rate (SMR; /pataka/), and Mandarin real-word SMR (怕它卡 /pa4 ta1 ka3/, 怕他看 /pa4 ta1 kan4/, 葡萄干 /pu2 tao2 gan1/). Item order was randomized within the AMR and real-word blocks. A brief /ka/ pretest was followed, for each formal stimulus, by one examiner demonstration and one unscored imitation; no additional formal practice was given. One formal production was planned per stimulus after a cue and deep breath. Repeats occurred only after a procedural or technical validity problem, not because a valid response was slow. At least 2 minutes separated blocks; approximately 3 minutes preceded a repeat.

采用固定区块顺序施测 7 个刺激：AMR（/pa/、/ta/、/ka/）、非词 SMR（/pataka/）及普通话真实词 SMR（怕它卡、怕他看、葡萄干）。AMR 及真实词区块内项目随机排序。简短/ka/预试后，每个正式刺激均先由检查者示范 1 次，再进行 1 次不计分模仿；不再安排额外正式练习。每个刺激计划正式完成 1 次，提示后深吸气开始。仅因程序或技术效度问题重复，不因有效反应较慢而重复。区块间休息至少 2 分钟；需要重做时约间隔 3 分钟。

**Audio interval and scoring review / 音频区间与评分复核**

To calculate correct oral-DDK rate (syllables/s), we multiplied credited tokens by the number of syllables per token and divided the result by the analyzed breath-trial duration. The interval began at the acoustic onset of the first credited production and ended at the offset of the last production in that breath trial; a pause longer than 0.2 seconds ended the current analysis interval. The demonstration and unscored imitation were excluded. For monosyllables, a token was credited when the target initial consonant was correct. Trisyllabic stimuli were credited only as fully correct whole tokens; no partial credit was assigned. Time occupied by uncredited productions after the first credited production was included in the analyzed duration.

正确 oral-DDK 速率（音节/秒）按以下方式计算：计入的正确词项数乘以每个词项的音节数，再除以一口气试次的分析区间时长。分析区间从首个可计分产出的声学起点开始，到该次一口气试次最后一个产出的声学终点结束；出现超过 0.2 秒的停顿即终止当前分析区间。示范和不计分模仿不纳入。单音节以目标声母正确为计入标准；三音节刺激只有完整词项全部正确才计入，不给予部分分。首个可计分产出之后，未计入产出所占的时间仍计入分析区间时长。

Two trained medical-student raters independently scored every recording by listening and waveform inspection. One speech-language pathologist (SLP) audited a random 10% as a third review. Training records documented task-level intraclass correlation coefficients (ICCs) above .90; analytic-sample agreement will be reported by task with 95% confidence intervals. By September 2026, scoring and verification of the oral-DDK recordings had been completed.

2 名接受培训的医学生评分者结合听辨与波形检查独立评分每段录音；1 名言语语言治疗师（SLP）随机抽取 10%作第三次复核。培训记录显示任务层面的组内相关系数（ICC）均高于 0.90；分析样本的一致性将按任务报告 95%置信区间。截至 2026 年 9 月，oral-DDK 录音的评分与复核已完成。
