# Supplementary material for: Multi-Informant Oral Diadochokinesis Assessment in Mandarin-Speaking Children and Adolescents: Protocol for a Cross-Sectional Observational Study
Source: JMIR Res Protoc. 2026 Nov 3;15:e99571. doi: 10.2196/99571 (PMC13730412; doi:10.2196/99571)
Supplement: Supplementary file 5 [file resprot-v15-e99571-s005.pdf]

STROBE Checklist for the Observational Study

The Strengthening the Reporting of Observational Studies in Epidemiology (STROBE) checklist [1] identifies where the manuscript reports the implemented procedures, participant flow, analysis approach, and dated study status. The primary rate analyses were complete by September 2026, but their inferential results are not included in this protocol. Parent/teacher association analyses are identified as not yet begun.

Table S1. STROBE checklist

Item	STROBE recommendation	Location or response in revised manuscript
1a	Indicate study design in title or abstract	Title and Abstract identify the manuscript as a protocol for a cross-sectional observational study.
1b	Provide an informative and balanced summary	Structured Abstract: Background, Objective, Methods, Results, Conclusions.
2	Explain scientific background and rationale	Introduction: conventional oral diadochokinesis (oral-DDK), the mainland Mandarin pediatric real-word evidence gap, functioning and child-development frameworks, and completed formative work.
3	State specific objectives, including hypotheses	Introduction ending: correct-rate task comparisons and source-specific contextual questions; readiness and five-action screening determine eligibility.
4	Present key elements of study design	Methods: Study Design and Protocol Status.
5	Describe setting, locations, and relevant dates	Methods: Setting and Participants; Results: recruitment and fieldwork dates and study status as of September 2026.
6	Describe participants and eligibility criteria	Methods—Setting and Participants: schools selected at random from the citywide sampling frame; all classes included and all eligible students invited; Putonghua-only everyday communication was the operational language criterion.
7	Clearly define outcomes, exposures, predictors, confounders	Methods; Tables 1-2; Multimedia Appendices 1-3: test-day health items; measured height and weight and calculated body mass index (BMI); forced vital capacity (FVC) measured twice standing; readiness and five-action eligibility rules; 3 parent and 3 teacher past-month items analyzed separately.

Item	STROBE recommendation	Location or response in revised manuscript
8	Describe sources of data and measurement methods	Methods and Multimedia Appendices 3-4: study-specific, self-developed readiness materials with fixed 5W1H question types, administration and scoring rules; actual item materials are not reproduced. Seven-stimulus oral-DDK battery, fixed block order, within-block randomization, practice and repeat rules, correct-token counting, acoustic interval boundaries and pause >0.2 seconds termination, recording files, dual scoring, and 10% speech-language pathologist (SLP) audit.
9	Describe efforts to address bias	Methods: 12-hour item-by-item training, a separate 100-student practice sample, dual scoring, random 10% SLP audit, child-level readiness/oromotor eligibility, and task-level technical audio checks. Discussion addresses residual selection and contextual-report measurement limitations.
10	Explain study size	Methods—Sample Size Justification: the doctoral proposal recruitment calculation and attrition allowance explain the recruitment target. This calculation does not establish power for the current repeated-task mixed-effects analyses; that limitation is stated.
11	Explain handling of quantitative variables	Methods and Table 2: core mixed-effects correct-rate model; readiness/oromotor checks used for eligibility, not as predictors among children who passed; separate health, anthropometric, FVC, and parent/teacher rate-direction comparisons. Frequency ratings are ordered categories.
12a	Describe all statistical methods	Methods—Statistical Analysis Plan and Table 2: core model terms, source-specific comparisons, item-level agreement, missing-data handling, ordinal association models with conditional rank-based sensitivity analysis, and within-family false-discovery-rate control. Associations are reported with uncertainty regardless of direction.
12b	Describe methods to examine subgroups/interactions	Statistical Analysis Plan: task, centered age, sex, all 2-way interactions, and the 3-way interaction; physician records tested separately; mutually exclusive fast/slow observations tested against adjusted correct rate.
12c	Explain how missing data will be addressed	Statistical Analysis Plan: usable denominators reported for each model; missing or technically unusable task records not imputed and described by stimulus, age band, sex, and reason; remaining missingness reported by source. Contradictory fast/slow ratings affect rate-direction analyses only.

Item	STROBE recommendation	Location or response in revised manuscript
12d	Cohort/case-control/cross-sectional-specific methods	Cross-sectional protocol: repeated tasks nested within participants; school recruitment, eligibility, respondent qualifications, and source-specific comparisons described. The scope of generalization is addressed in Strengths and Limitations.
12e	Describe sensitivity analyses	Methods—Statistical Analysis Plan and Parent and Teacher Contextual Reports: height with weight compared with BMI alone; ordinal-model assumption check and conditional rank-based sensitivity analysis. If an item has no category variation, its association with rate is reported as not estimable rather than as zero.
13	Report participant flow	Results and Figure 1: 1,036 entered fieldwork; 2 withdrew, 6 had technical failures, and 2 could not complete required procedures; 10 did not complete and 1,026 were retained.
14	Report descriptive data	Results: recruited, noncompletion, and retained counts; final baseline characteristics will accompany the inferential results.
15	Report outcome data	Methods and Multimedia Appendix 4 specify outcome derivation. Recording scoring and verification were complete by September 2026; outcome estimates are not reported in this protocol.
16	Report main results	Results: primary task-, age-, and sex-related rate analyses were complete by September 2026. The normative-rate manuscript was submitted and awaiting an editorial decision; its results are not included here.
17	Report other analyses	Results: parent/teacher rate-association analyses had not begun by September 2026. The same-cohort maximum phonation time report was published in August 2026; it does not report FVC-rate associations. Subsequent accuracy and acoustic work remains separate.
18	Summarize key results	Discussion—Principal Features of the Protocol and Relation to Prior Work: implemented procedures and the questions they support. Final inferential findings are outside this protocol report.
19	Discuss limitations	Discussion—Strengths and Limitations: one-region school cohort and selection; task-specific denominators; limited validation and no contextual-report retest in this cohort; item-level limits to exact replication of the readiness check; lack of an independent minimal-record comparator; and limits of the recruitment calculation for current model power.

Item	STROBE recommendation	Location or response in revised manuscript
20	Give cautious interpretation	Discussion and Conclusions: task comparisons do not establish superiority of the complete protocol; contextual associations remain empirical questions. No informant or combined score is treated as a diagnostic standard.
21	Discuss generalizability	Discussion: Mandarin-speaking students from mainstream schools in one region; use in atypical or clinical populations remains to be tested.
22	Give funding source and funder role	Funding section included.

References

1. von Elm E, Altman DG, Egger M, Pocock SJ, Gøtzsche PC, Vandenbroucke JP; STROBE Initiative. The Strengthening the Reporting of Observational Studies in Epidemiology (STROBE) statement: guidelines for reporting observational studies. PLoS Med. 2007;4(10):e296. doi:10.1371/journal.pmed.0040296
